# Sp110 transcription is induced and required by *Anaplasma phagocytophilum *for infection of human promyelocytic cells

**DOI:** 10.1186/1471-2334-7-110

**Published:** 2007-09-20

**Authors:** José de la Fuente, Raúl Manzano-Roman, Edmour F Blouin, Victoria Naranjo, Katherine M Kocan

**Affiliations:** 1 Department of Veterinary Pathobiology, Center for Veterinary Health Sciences, Oklahoma State University, Stillwater, OK 74078, USA; 2 Instituto de Investigación en Recursos Cinegéticos IREC (CSIC-UCLM-JCCM), Ronda de Toledo s/n, 13071 Ciudad Real, Spain

## Abstract

**Background:**

The tick-borne intracellular pathogen, *Anaplasma phagocytophilum *(Rickettsiales: Anaplasmataceae) causes human granulocytic anaplasmosis after infection of polymorphonuclear leucocytes. The human Sp110 gene is a member of the nuclear body (NB) components that functions as a nuclear hormone receptor transcriptional coactivator and plays an important role in immunoprotective mechanisms against pathogens in humans. In this research, we hypothesized that Sp110 may be involved in the infection of human promyelocytic HL-60 cells with *A. phagocytophilum*.

**Methods:**

The human Sp110 and *A. phagocytophilum msp4 *mRNA levels were evaluated by real-time RT-PCR in infected human HL-60 cells sampled at 0, 12, 24, 48, 72 and 96 hours post-infection. The effect of Sp110 expression on *A. phagocytophilum *infection was determined by RNA interference (RNAi). The expression of Sp110 was silenced in HL-60 cells by RNAi using pre-designed siRNAs using the Nucleofector 96-well shuttle system (Amaxa Biosystems, Gaithersburg, MD, USA). The *A. phagocytophilum *infection levels were evaluated in HL-60 cells after RNAi by real-time PCR of *msp4 *and normalizing against human *Alu *sequences.

**Results:**

While Sp110 mRNA levels increased concurrently with *A. phagocytophilum *infections in HL-60 cells, the silencing of Sp110 expression by RNA interference resulted in decreased infection levels.

**Conclusion:**

These results demonstrated that Sp110 expression is required for *A. phagocytophilum *infection and multiplication in HL-60 cells, and suggest a previously undescribed mechanism by which *A. phagocytophilum *modulates Sp110 mRNA levels to facilitate establishment of infection of human HL-60 cells.

## Background

*Anaplasma phagocytophilum *(Rickettsiales: Anaplasmataceae) is an obligate intracellular tick-borne pathogen that causes human granulocytic anaplasmosis (HGA), tick-borne fever of ruminants, and equine and canine granulocytic anaplasmosis [1]. HGA, first described in 1994 in the United States, has become a predominant form of anaplasmosis and among the most common tick-borne pathogens in the United States and Europe [2]. HGA is characterized by fever, headache, myalgia, and malaise, as well as leukopenia, thrombocytopenia, and elevated levels of C-reactive protein and liver transaminases, which are indicators of inflammatory response and hepatic injury, respectively [2]. Although the disease is usually self-limiting, severe complications can result, including prolonged fever, shock, seizures, pneumonitis, acute renal failure, hemorrhage, rhabdomyolysis, opportunistic infections and death [2].

*A. phagocytophilum *initiates infection of polymorphonuclear leucocytes by adhesion to host cells, a process which involves adhesins, such as the human P-selectin glycoprotein ligand-1 **(**PSGL-1) that bind cooperatively to neutrophil ligand molecules [2]. After infection, *A. phagocytophilum *undergoes a developmental cycle in parasitophorous vacuoles that includes reticulated and dense forms, and this infection modulates host cell growth and differentiation [3].

While the main vector for *A. phagocytophilum *are tick species belonging to the *Ixodes ricinus *complex, the pathogen multiplies in a broad range of terrestrial vertebrates [2,4]. In the laboratory, *A. phagocytophilum *can be propagated in undifferentiated human promyelocytic HL-60 cells. Infection of HL-60 cells with *A. phagocytophilum *results in modulation of host cell gene expression (see for example [5,6].

Sp110 is a member of the nuclear body (NB) components that functions as a nuclear hormone receptor transcriptional coactivator [7]. Sp110 and other NB-associated proteins, induced by type I (α/β) and type II (γ) interferons (IFNs), play a role in IFN response and virus replication [8]. Sp110 expression is induced in human peripheral blood leukocytes and spleen but not in other tissues [8]. Sp110 inhibits vesicular stomatitis virus and influenza virus replication, confers resistance to human Foamy virus, and gene polymorphisms or mutations have been associated with susceptibility to the Hepatitis C virus and immunodeficiency and hepatic veno-occlusive disease [8-10].

Recently, the mouse Sp110 homologue, the intracellular pathogen resistance 1 (*Ipr1*) gene, was shown to control susceptibility to *Mycobacterium tuberculosis *in mice [11]. As in mice, *Ipr1*-like expression was higher in European wild boar resistant to natural *M. bovis *infection [12]. Pan et al. [11] proposed that Ipr1-related proteins may play a role in integrating signals generated by intracellular pathogens or viruses with host cell mechanisms that regulate gene expression and cell death, thus modulating host susceptibility to infection. However, recent publications have documented that polymorphisms in Sp110 gene are not associated with susceptibility to tuberculosis in humans [13-15]. These results suggest that Sp110 may have a different role during infection by intracellular bacterial pathogens in humans.

In the study reported herein, we hypothesized that Sp110 may be involved in the infection of human promyelocytic cells with *A. phagocytophilum *and used a combination of real-time RT-PCR and RNA interference (RNAi) to test this hypothesis.

## Methods

### Determination of Sp110 mRNA levels in uninfected and infected HL-60 cells

Human HL-60 cells were cultured and infected with *A. phagocytophilum *as previously described (multiplicity of infection, MOI = 2) [5]. Uninfected and infected cultures were sampled at 0, 12, 24, 48, 72 and 96 hours post-infection (hpi) and Sp110 and major surface protein 4 (*msp4*) mRNA levels were determined by real-time RT-PCR using human Sp110 (Genbank accession number NM_004509) and *msp4 *[4] sequence-specific primers (Sp110, forward: 5'-cttcctatgaacggcagagc; reverse: 5'-ggcgactcactcaggatctc; *msp4*, APMSP4RT5: 5'-tgacaggggaggatcttacg and APMSP4RT3: 5'-tctagctccgccaatagcat) and the QuantiTec SYBR Green RT-PCR kit (Qiagen, Valencia, CA, USA) in a Bio-Rad iQ5 thermal cycler (Hercules, CA, USA) following manufacturer's recommendations. mRNA levels were normalized against human β-actin (forward: 5'-tgatatcgccgcgctcgtcgtc; reverse: 5'-gccgatccacacggagtact) [5] and displayed in mRNA arbitrary units. Sp110 mRNA levels were compared between infected and uninfected cells by ANOVA test (P = 0.05).

### RNA interference in HL-60 cells

**Figure 1:**
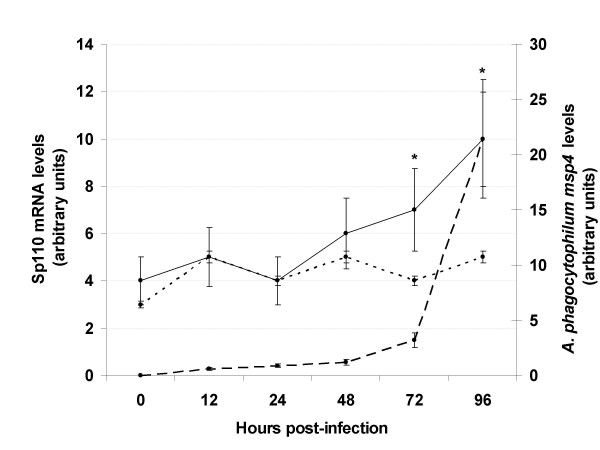
Expression kinetics of Sp110 in human HL-60 cells infected with *A. phagocytophilum*. Human Sp110 (solid and dotted lines) and *A. phagocytophilum msp4 *(broken line) mRNA levels were determined by real-time RT-PCR in uninfected and infected HL-60 cells. mRNA levels were normalized against human β-actin and displayed in mRNA arbitrary units. Sp110 mRNA levels were compared between infected (solid line) and uninfected (dotted line) cells using an ANOVA test (*P < 0.05; N = 3).

The expression of Sp110 was silenced in HL-60 cells by RNAi using a combination of two different pre-designed siRNAs to Sp110 (siRNAs IDs 145432 and 241448) and to actin-related protein 3 (ARP3) (NM_020445; siRNAs IDs 127242 and 127243) and P-selectin glycoprotein ligand-1 (PSGL-1) (NM_003006; siRNAs IDs 12441 and 142575) as negative and positive controls, respectively (Ambion, Austin, TX, USA). In a 96-well plate, 4 × 10^5 ^cells/well were nucleofected with 1 μg of siRNA using the Nucleofector 96-well shuttle system (Amaxa Biosystems, Gaithersburg, MD, USA) with kit SF and program 96-EN-138 following manufacturer's instructions (efficiency of transfection, 87 ± 13% after 24 hours). After nucleofection, cells were divided into two 96-well plates. Twenty four hours after nucleofection, cells were collected from one plate for cell viability and morphology assessment of Giemsa-stained cytospin smears and RNA extraction (RNeasy 96 kit, Qiagen) and analysis of gene expression by real-time RT-PCR as described above. The second plate was incubated for 24 hours with cell-free *A. phagocytophilum *(MOI = 10) prepared as described by Thomas and Fikrig [16]. This level of infection corresponds to approximately 72 hpi in Figure 1. The cells were then washed 3× with PBS and total DNA was extracted (Wizard SV 96 genomic DNA purification system, Promega, Madison, WI, USA). The *A. phagocytophilum *infection levels were evaluated in HL-60 cells after RNAi by real-time PCR of *msp4 *and normalizing against human *Alu *sequences [17] using the QuantiTec SYBR Green PCR kit (Qiagen) in an iQ5 thermal cycler (Bio-Rad) as described above. Known amounts of the full length *A. phagocytophilum msp4 *PCR were used to construct a standard curve for the real-time PCR. Sp110 and PSGL-1 mRNA levels were determined after RNAi by real-time RT-PCR, normalized against human β-actin using the comparative Ct (delta delta Ct) method and compared between Sp110 or PSGL-1 siRNA- and ARP3 siRNA-treated control cells by Student's t-Test (P = 0.05). *A. phagocytophilum msp4 *DNA levels were compared between cells nucleofected with Sp110 or PSGL-1 siRNAs and control cells treated with ARP3 siRNA by Student's t-Test (P = 0.05).

## Results and discussion

The Sp110 mRNA levels increased in HL-60 cells after 24 hpi with *A. phagocytophilum *and reached 2× induction at 96 hpi (Fig. 1). The increase in Sp110 mRNA levels coincided with pathogen multiplication and increasing infections (Fig. 1) and may reflect a protective cellular response to limit rickettsial infection or the result of the manipulation by *A. phagocytophilum *of host gene transcription to promote pathogen multiplication.

**Figure 2:**
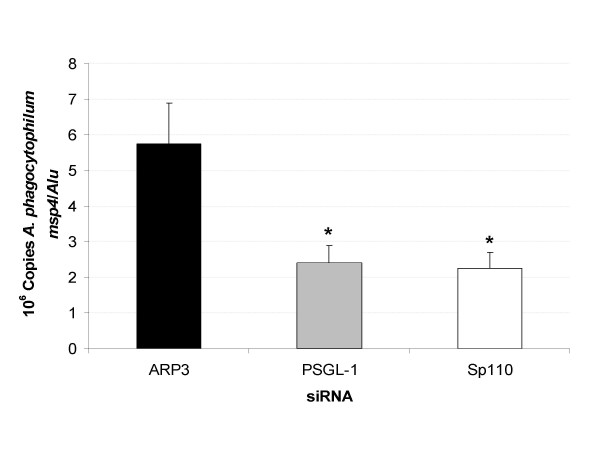
Effect of Sp110 on *A. phagocytophilum *infection of human HL-60 cells. *A. phagocytophilum msp4 *DNA levels were determined by real-time PCR in infected HL-60 cells with knockdown Sp110, PSGL-1 (positive control) and ARP3 (negative control). *A. phagocytophilum msp4 *DNA levels were normalized against human *Alu *sequences and infection levels were compared between cells nucleofected with Sp110 or PSGL-1 siRNAs and control cells treated with ARP3 siRNA by Student's t-Test (*P < 0.05; N = 8).

To test these hypotheses, the effect of Sp110 silencing by RNAi was evaluated on *A. phagocytophilum *infection of HL-60 cells. Twenty four hours after RNAi, 63 ± 1% cells were viable and cell morphology was not affected in all treatments. Furthermore, the expression of Sp110 and PSGL-1 were shown to be silenced by 85 ± 9% and 61 ± 18% (P < 0.05; N = 8), respectively when compared to ARP3 siRNA-nucleofected controls. After RNAi, cells were infected with *A. phagocytophilum *at a MOI equivalent to approximately 72 hpi in Figure 1. The results of RNAi showed a reduction in *A. phagocytophilum *DNA in cells nucleofected with Sp110 siRNA, similar to results obtained in positive control cells transfected with PSGL-1 siRNA (Fig. 2). If Sp110 has a role in the control of *A. phagocytophilum *infection in humans, we would have expected higher infection levels in HL-60 cells with knockdown Sp110. However, although Sp110 protein levels were not determined, the results of RNAi suggested that Sp110 was required for *A. phagocytophilum *infection and/or multiplication in HL-60 cells.

The studies of Sp110 function demonstrated that this protein has an important role in immunoprotective mechanisms against pathogens in humans [9]. However, Sp110 is also used by some viruses such as Epstein-Barr virus, to enhance replication in infected cells [18]. As shown here for *A. phagocytophilum*, Sp110 transcription is induced by some DNA viruses, suggesting that this mechanism may represent an evolutionary adaptation that facilitates pathogen replication [18].

## Conclusion

In summary, we have shown that *A. phagocytophilum *increases Sp110 mRNA levels in infected human promyelocytic HL-60 cells. These results suggest a new mechanism by which *A. phagocytophilum *modulates gene expression through NB-associated proteins. Furthermore, silencing of Sp110 expression reduced pathogen infection/multiplication, thus suggesting that *A. phagocytophilum *can modulate the transcription of Sp110 to facilitate infection of human HL-60 cells. The mechanism by which *A. phagocytophilum *modulates the transcription of Sp110 to enhance infection of human HL-60 cells will require further study.

## Competing interests

The author(s) declare that they have no competing interests.

## Authors' contributions

JF and KMK conceived and designed the experiments and wrote the paper. JF analyzed the data. RM-R, EFB, and VN performed the experiments. All authors have read and approved the final manuscript

## Pre-publication history

The pre-publication history for this paper can be accessed here:


